# Single cell cultures of *Drosophila *neuroectodermal and mesectodermal central nervous system progenitors reveal different degrees of developmental autonomy

**DOI:** 10.1186/1749-8104-4-30

**Published:** 2009-08-03

**Authors:** Karin Lüer, Gerhard M Technau

**Affiliations:** 1 Institute of Genetics, University of Mainz, 55099 Mainz, Germany

## Abstract

**Background:**

The *Drosophila *embryonic central nervous system (CNS) develops from two sets of progenitor cells, neuroblasts and ventral midline progenitors, which behave differently in many respects. Neuroblasts derive from the neurogenic region of the ectoderm and form the lateral parts of the CNS. Ventral midline precursors are formed by two rows of mesectodermal cells and build the CNS midline. There is plenty of evidence that individual identities are conferred to precursor cells by positional information in the ectoderm. It is unclear, however, how far the precursors can maintain their identities and developmental properties in the absence of normal external signals.

**Results:**

To separate the respective contributions of autonomous properties versus extrinsic signals during their further development, we isolated individual midline precursors and neuroectodermal precursors at the pre-mitotic gastrula stage, traced their development *in vitro*, and analyzed the characteristics of their lineages in comparison with those described for the embryo. Although individually cultured mesectodermal cells exhibit basic characteristics of CNS midline progenitors, the clones produced by these progenitors differ from their *in situ *counterparts with regard to cell numbers, expression of molecular markers, and the separation of neuronal and glial fate. In contrast, clones derived from individually cultured precursors taken from specific dorsoventral zones of the neuroectoderm develop striking similarities to the lineages of neuroblasts that normally delaminate from these zones and develop *in situ*.

**Conclusion:**

This *in vitro *analysis allows for the first time a comparison of the developmental capacities *in situ *and *in vitro *of individual neural precursors of defined spatial and temporal origin. The data reveal that cells isolated at the pre-mitotic and pre-delamination stage express characteristics of the progenitor type appropriate to their site of origin in the embryo. However, presumptive neuroblasts, once specified in the neuroectoderm, exhibit a higher degree of autonomy regarding generation of their lineages compared to mesectodermal midline progenitors.

## Background

The central nervous system (CNS) represents the organ with the highest structural complexity and cellular diversity. Normal function of the CNS requires the generation of specific types and numbers of neuronal and glial cells during development following a reproducible spatio-temporal programme. Accordingly, the process conferring individual identities and properties to neural stem cells is fundamental and is a major issue in developmental neurobiology. The availability of a broad range of molecular and genetic tools as well as micromanipulation techniques have made *Drosophila *a suitable model organism to study this process at the level of individually identifiable cells.

The *Drosophila *CNS develops from two different populations of progenitor cells. Comprising the vast majority of neural precursors, the neuroblasts (NBs; about 30 per truncal hemisegment) generate the prominent lateral parts of the CNS. They delaminate individually in a specific spatio-temporal pattern from the neurogenic region of the ectoderm after a process of lateral inhibition that separates them from presumptive epidermoblasts. The second group of progenitor cells gives rise to the CNS midline. They are located ventrally between the neuroectoderm and the mesodermal primordium as one continuous row of cells on either side (three to four cells per hemisegment), which meet at the ventral midline upon invagination of the mesoderm during gastrulation. All of these mesectodermal cells become CNS midline progenitors.

Generally, NBs act like stem cells, generating a number of secondary precursors (called ganglion mother cells (GMCs)) by asymmetric divisions, which normally divide once to produce two post-mitotic progeny. Each of the NBs assumes an individual identity, as reflected by the expression of a specific combination of molecular markers [1,2] and the generation of a specific cell lineage [3-5]. Specification of the individual NB fates occurs in the ectoderm based on positional information provided by the products of segment polarity genes [6], dorsoventral patterning genes [7], homeotic genes [8], and temporal cues [9]. The respective developmental traits conferred by these factors become manifested in neuroectodermal progenitor cells to different degrees [9-11]. Although most of the factors controlling specification of presumptive NBs appear to act in the neuroectoderm, it is still an open question whether NBs upon delamination from the neuroectoderm express their specific fate autonomously or whether they require inductive signals from surrounding tissues. It has been recently shown by *in vitro *culture experiments that embryonic NBs require extrinsic signals from the overlying epithelium for orientation of their division axis [12]. However, as to how far these or other extrinsic signals are required for the expression of further characteristics or the maintenance of NB fate and, thus, for the production of their characteristic lineages is unknown.

The second set of progenitor cells, the CNS midline progenitors, behave differently from NBs in many respects [13-15]. Similar to the floor plate in vertebrates, the *Drosophila *ventral midline acts as an organizing centre, as it influences cell fate in the lateral CNS [16-20], and is essential for proper organization of the axonal network [21]. In contrast to the NBs, the segmental number of mesectodermal midline progenitors is variable (six to eight cells per segment) [22]. During embryogenesis they give rise to about 20 functionally diverse cells, including interneurons, motoneurons and glial cells belonging to five different types of lineages (four neuronal and one glial type) [22]. Except for the median NB (MNB), which divides in a stem cell mode, midline progentior cells (with regard to geometry) perform equal divisions. A large number of genes have been found to be expressed in the midline, and individual precursors and progeny cells differ by the combinations of genes they express, reflecting a high degree of cellular diversity [15,23-25]. It has been recently shown that several aspects of midline cell fate become determined after division of the precursors by intercellular communication among progeny cells involving Wingless, Hedgehog [23] and Notch [15] signalling. Here we ask to what extent pre-mitotic midline progenitor cells (similar to NBs; see above) require positional cues and/or early inductive signals in the (mes)ectoderm for normal specification.

In order to investigate the dependency of the two sets of CNS progenitor cells on extrinsic versus intrinsic signals, we assayed their cell-autonomous developmental capabilities. We developed a strategy to remove cells from specific positions in embryos shortly after onset of gastrulation and growing them individually in culture. At the early gastrula stage, ectodermal precursor cells have just completed cellularization and have not yet entered postblastodermal mitosis, and their positions can be precisely determined due to the presence of first morphological landmarks. Since the normal lineages of all CNS midline precursors [22] and all truncal NBs, as well as their sites of origin in the neuroectoderm, are known [3-5], a direct comparison between the cultured clones and specific neural lineages *in situ *was possible.

The development of cultured neuroectodermal and midline progenitors was traced using time-lapse analysis and cell-specific molecular markers. We find that clones produced by cultured midline precursors differ from their *in situ *counterparts with regard to cell numbers and the expression of neuronal versus glial markers, whereas cultured neuroectodermal precursors develop striking similarities to specific NB lineages *in situ *with regard to division pattern, clone size and marker gene expression.

## Results

### Development of mesectodermal midline progenitors in single cell cultures

**Figure 1:**
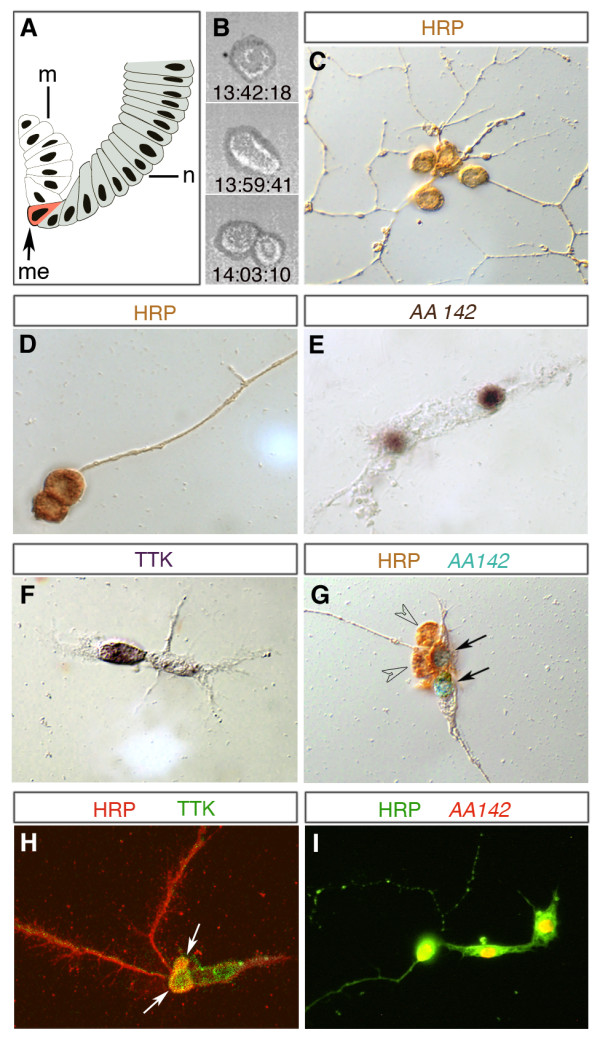
**Clones derived from individually cultured mesectodermal midline progenitors**. **(A) **Schematic cross-section showing one side of the ventral half of an embryo at stage 7 (early gastrula stage; midline to the left, dorsal to the top). The row of mesectodermal midline progenitors (me; marked in red) is located at the ventral furrow between the neuroectoderm (n; grey) and the invaginated mesoderm (m; white). **(B) **Three frames from a time-lapse recording (real time is indicated) showing morphologically symmetrical division of an isolated midline progenitor. **(C-I) **Individually cultured midline progenitors give rise to clones that, based on morphological criteria, consist of neuronal (C,D) or glial cells (E,F) or a mixture of both (G-I). In (C,D) the entire lineage stains positive for the neuronal marker anti-horse radish peroxidase (HRP), and cells in (E) for the midline glia marker AA142-*lacZ *(nuclear staining). In (F) only one of the two cells is labelled by the midline glia marker anti-Tramtrack (TTK). The clone in (G) comprises two anti-HRP-positive (brown; arrowheads) and two AA142-positive cells (blue nuclei; arrows)). (H,I) Lineages including cells that co-express glial and neuronal markers. The clone in (H) consists of four TTK (green) expressing cells, two of which (yellow; arrows) co-express HRP (red); the clone in (I) consists of three cells in which AA142-*lacZ *(red) and HRP (green) are co-expressed (yellow). Markers applied in (C-I) are indicated. The size of midline progenitor cells is approximately 12 μm and of progeny cells 5 to 8 μm.

Cells were removed from the ventral midline at embryonic stage 7/8 upon invagination of the mesodermal primordium (Figure 1A) when the two rows of mesectodermal progenitor cells are facing each other and become clearly distinguishable at both sides of the ventral furrow. At this stage midline progenitor cells have not yet entered mitosis. Single progenitor cells were transferred into the culture medium and allowed to develop further for 16 to 20 hours, which corresponds to the time required for normal embryos to fully develop (stage 16/17).

#### Distribution of clone sizes

Each of the cultured midline precursors (n = 896) gave rise to two to four progeny cells (Figure 1B–I); 37% (n = 331) of their clones consisted of two cells, 19% (n = 173) of three cells, and 44% (n = 392) of four cells. We never found clones consisting of more than four cells. This significantly differs from the situation *in situ *[22], in which about 75% of DiI-labelled midline clones (at stage 16/17) consisted of only 2 cells (including the MP1, UMI, midline glia, and most of the VUM clones), about 7% comprised 4 cells (15% of the VUM clones, and a few mixed lineages; see below), and about 18% comprised 5 to 8 cells (including the MNB and 6% of the VUM clones). Midline clones consisting of three cells are very rare exceptions *in vivo *(Table 1).

**Table 1:** Developmental properties of mesectodermal midline progenitors developing *in vivo *and in single cell cultures

	In vivo*	*In vitro*^†^
Division pattern		
Symmetric	87%	100%
Asymmetric	13%	-
		
Clone size		
2 cells	74.3%	37%
3 cells	0.5%	19%
4 cells	7.6%	44%
5–8 cells	17.5%	-
		
Morphology		
Neuronal	84.5%	78.4%
Glial	14.5%	4.4%
Mixed	1%	17.2%
		
Co-expression		
HRP+TTK	-	+
HRP+AA142	-	+
ODD+AA142	-	+
		
Cell death	+	+

*In vivo data according to [22]. ^†^For number of cases (n) see text.

#### Division pattern

Using time-lapse recordings, we traced the division patterns of cultured midline precursor cells (n = 41). In all cases the divisions were morphologically symmetrical (Figure 1B). The first division takes place 40 to 55 minutes after gastrulation (when cells were taken into culture), which is similar to the *in vivo *situation (about 40 minutes) [22]. Subsequently, one (three-cell clones) or both daughter cells (four-cell clones) may divide one more times within 3 to 7 hours after gastrulation. In the embryo, NBs and one of the midline precursors, the MNB, divide asymmetrically several times to self renew. We never found such a stem cell mode of division for isolated midline precursors *in vitro*.

#### Differentiation

As judged by morphological criteria, cultured midline cells differentiated into neuronal and glial cell types. Neuronal cells showed small spherical cell bodies and developed long fibres that project in various directions (Figure 1C) or fasciculate with each other (Figure 1D). Glial cells typically show flat and elongated cytoplasmic extensions (Figure 1E). According to these criteria, 4.4% of the clones were glial and 78.4% neuronal. In addition 17.2% of the clones appeared to consist of both types of cells (Figure 1G–I). In the embryo, six midline precursors per segment (one MP1, one UMI, one MNB, three VUM; for different nomenclature see [15]) generate neurons exclusively, and one to three precursors (the exact number is unknown) produce only glia. Midline glia and neurons normally share common lineages only in exceptional cases (about 1% of DiI labelled clones were compound MP1/midline glia clones [22]; Table 1).

The existence of mixed neuronal/glial clones *in vitro *is further supported by cell-specific molecular markers. Applying the neuronal marker anti-horse radish peroxidase (anti-HRP; n = 12), clones were entirely labelled in only 50% of the cases (Figure 1C, D), whereas labelled and unlabelled cells coincide in the other 50%. Similar observations were made using anti-Tramtrack (TTK) antibodies (n = 50; Figure 1F, H) and the enhancer-trap line AA142 (n = 188; Figure 1E, G, I) as markers for midline glia [14,26,27]. These led to partially labelled clones in 46% (n = 23; Figure 1F)) and 37% (n = 70) of cases, respectively. Upon double labelling with both midline glia markers, AA142 and anti-TTK, we found several cases (n = 10) in which the individual progeny cells expressed only one of these markers (data not shown).

Next, we performed double labelling against anti-HRP and anti-TTK or AA142. Out of 45 clones, 30 were positive for one, and 15 were positive for both markers (Figure 1G–I). Interestingly, most of the latter ones included cells that co-expressed both markers. For example, the clone shown in Figure 1H consists of four cells, two of which are positive for both anti-TTK and anti-HRP. The clone in Figure 1I consists of three cells, all of which coexpress anti-HRP and AA142. Furthermore, the transcription factor Odd-skipped (anti-ODD), which normally specifically labels the MP1 neurons [25], is found to be co-expressed with AA142 in individually cultured clones (n = 8; not shown). Instability in the separation and/or maintenance of glial versus neuronal fate in cultured midline lineages is further supported by structural dynamics, as revealed by time-lapse recordings: during late stages of differentiation some cells changed their shape significantly and appeared to convert from glial to neuronal morphology (Additional file 1).

#### Apoptosis

**Figure 2:**
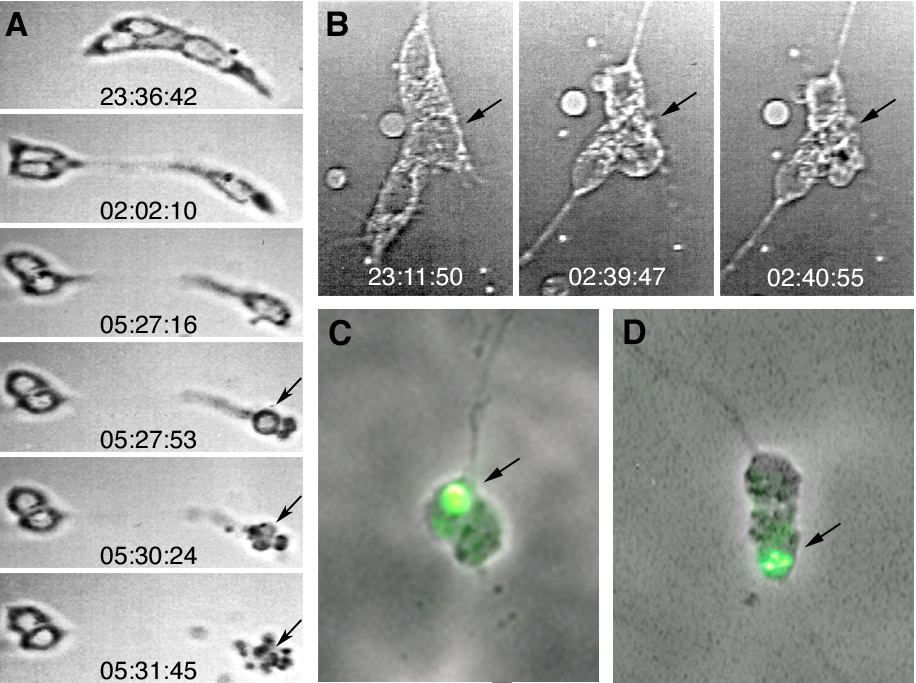
**Programmed cell death occurring in individually cultured midline lineages**. **(A,B) **Frames from time-lapse recordings (real time is indicated) of two developing midline clones (see Additional files 1 and 2 for the corresponding movies). In both clones one out of three cells disintegrates into smaller particles within a short period of time (arrows). The cell in (A) moves apart from its siblings before undergoing apoptosis. Migration over such distances in neural cell cultures is normally only performed by glial cells. **(C,D) **Midline clones in which apoptosis of one cell is indicated by TUNEL staining (arrow). Cell sizes correspond to 5 to 8 μm.

Programmed cell death has been reported to occur in the embryonic CNS midline, especially in the glial lineages as revealed by marker gene expression in embryos deficient for apoptosis [14,28,29]. Accordingly, in such embryos (*Df(3L)H99*, lacking the three key genes for the induction of apoptosis, *reaper*, *hid*, and *grim *[30]) midline glial cell clones labelled with DiI consisted of four to six cells at stage 17 (Janina Seibert and GMT, unpublished observations) instead of only two in the wild type. To examine whether the apoptotic cell fate is also expressed by midline clones growing in isolation, we first looked for morphological indications. In several cases (11 out of approximately 50 cases), between 2.5 and 14 hours after the progenitor was taken into culture (stage 7/8), we observed individual progeny cells that rounded up and finally disintegrated into smaller particles. Morphological changes from first rounding until fragmentation of the cell (Figure 2A, B) take no more than 25 minutes as revealed by time-lapse video microscopy (Additional files 1 and 2). The occurrence of apoptosis in some of the cultured midline clones is further indicated by TUNEL-staining (Figure 2C, D). Thus, midline clones grown in isolation do express the apoptotic cell fate. However, our data do not allow drawing conclusions about the numbers and identities of the dying cells in culture.

Taken together, cells of the mesectoderm isolated at the pre-mitotic gastrula stage and grown *in vitro *exhibit some general characteristics of CNS midline progenitors. They divide symmetrically and give rise to progeny cells that express morphological characteristics of neuronal and glial cells or undergo programmed cell death. However, the distribution of clone sizes, the structural dynamics and (mis)expression of molecular markers indicate that their differentiation significantly differs from midline lineages developing *in situ *(summarized in Table 1). This suggests that midline cells are not dedicated to a particular fate at the precursor stage (stage 7), and are unable to generate a specific lineage autonomously. Instead, inductive signals appear to be required for the specification of the various midline cell types and/or maintenance of their fate.

### Development of progenitors from the ventral neuroectoderm in single cell culture

To analyse the developmental properties of isolated cells from the neuroectoderm, cells were removed from the ventral neurogenic region at the early gastrula stage (stage 7/8), prior to the onset of postblastodermal mitoses and to the delamination of NBs. Individual cells were immediately transferred to the culture medium and grown *in vitro *for up to 20 hours. Due to release from lateral inhibition, neuroectodermal cells grown in isolation produce neural clones exclusively [31].

#### Division patterns

**Figure 3:**
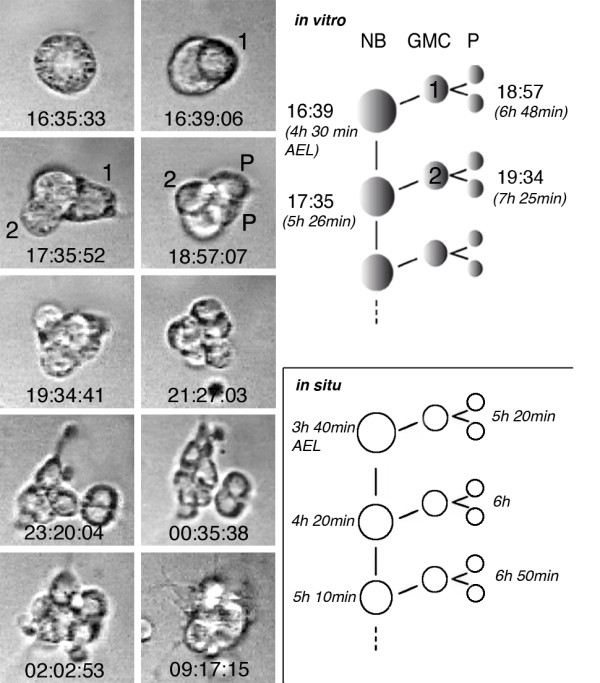
**Division pattern of an individually cultured neuroectodermal progenitor cell**. Left panel: selected frames from time-lapse recordings (real time is indicated) of a developing neuroectodermal progenitor cell (see Additional file 4 for the corresponding movie). Isolated neuroectodermal cells show a stem cell mode of division, which is typical for neuroblasts (NB). With each of their asymmetric divisions they self renew and bud off a smaller daughter cell (ganglion mother cell (GMC); the numbers 1 and 2, indicate the first two GMCs), which typically divides one more time into two postmitotic progeny (P). The size of the NB is approximately 10 μm and of postmitotic progeny cells 5 to 6 μm. Right panel: the time points when the first divisions took place in this particular lineage (grown at 22°C) are indicated at the top (compare left panel). The respective times after egg laying (AEL) are indicated in brackets. Note that the cell cycle of GMCs is significantly longer than that of the NB. The lower scheme shows for comparison the timing (at 25°C) of the first mitotic cycles of NBs and GMCs as observed *in situ *(according to Hartenstein *et al*. [69]).

Sizes of clones produced by the isolated neuroectodermal progenitors varied between 2 and more than 20 cells (exact counting of cell numbers is difficult in large clones). This is true for cultured clones derived from the ventral half, as well as those derived from the dorsal half of the neuroectoderm. The distribution of clone sizes corresponds to the situation *in situ *(Additional file 3). Also, the mode and timing of mitoses of the cultured progenitor cells correspond to the behaviour of NBs *in situ *(Figure 3; Additional file 4). Generally, they divide asymmetrically in a stem cell mode, budding off smaller daughter cells (GMCs), which divide one more time symmetrically. Cell cycles of GMCs are significantly longer than those of the NBs.

#### Differentiation

**Figure 4:**
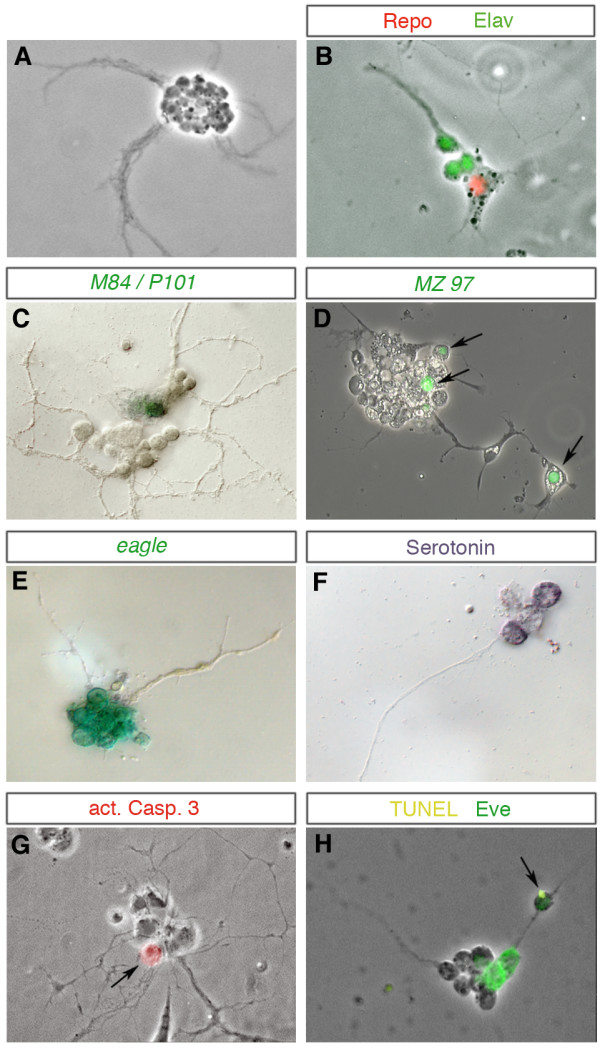
**Differentiation of individually cultured neuroectodermal progenitor cells**. **(A-H) **Cultured neuroectodermal progenitor cells produce between 2 and more than 20 neural progeny cells. Neurons show small round cell bodies and develop long fibre projections that sometimes fasciculate. Glia exhibit flat cytoplasmic protrusions with irregular shape. Besides general markers for neurons or glia (B) (anti-Elav, green; anti-Repo, red), some clones express markers characteristic for particular subsets of neurons ((F) anti-serotonin; (H) anti-Eve), for subsets of glial cells ((C) M84/P101-*lacZ*; (D) Mz97-GFP, arrows) or for particular lineages ((E) *eagle-lacZ*). Some progeny cells undergo programmed cell death as revealed by anti-activated Caspase 3 (G, arrow) or TUNEL staining (H) (see yellow spot (arrow) in distant cell of clone double-stained against Eve). Markers applied in (B-H) are indicated. The sizes of postmitotic progeny cells correspond to 5 to 6 μm.

Most of the clones consist of neurons exclusively, as judged by morphological criteria (Figure 4A). Some of the clones comprise glial cells in addition to neurons as confirmed by the expression of Repo (Figure 4B), a general marker for lateral glia cells [32]. We also observed expression of markers that are specific for smaller subsets of cells. For example, within some of the cultured clones individual cells express M84/P101-lacZ (Figure 4C) or MZ97-GFP (Figure 4D), which are specific to subsets of glial cells in the embryo, like the subperineurial glia [33]. While neuronal progeny stay in a dense cluster, glial cells sometimes move away from the cluster (Figure 4D), which also resembles the situation *in situ*. Several of the individually cultured neuroectodermal progenitor cells produced clones staining positive for Eagle (Figure 4E), expression of which is restricted to four lineages in the embryo (those of NBs 2–4, 3-3, 6-4 and 7-3 [34]). Furthermore, a few clones included one to two cells expressing the neurotransmitter serotonin (Figure 4F). In the embryo, serotonergic neurons derive exclusively from NB7-3; the late embryonic NB7-3 lineage consists of four cells, two of which express serotonin (as is the case for the clone shown in Figure 4F).

Programmed cell death is part of the normal developmental programme of most of the NB lineages [35]. Accordingly, using anti-activated Caspase 3 (Figure 4G) or TUNEL staining (Figure 4H), we detected apoptosis of individual neuronal progeny cells in some clones of cultured neuroectodermal progenitor cells.

#### Cultured progenitors generate specific types of lineages depending on their site of origin within the neuroectoderm

**Figure 5:**
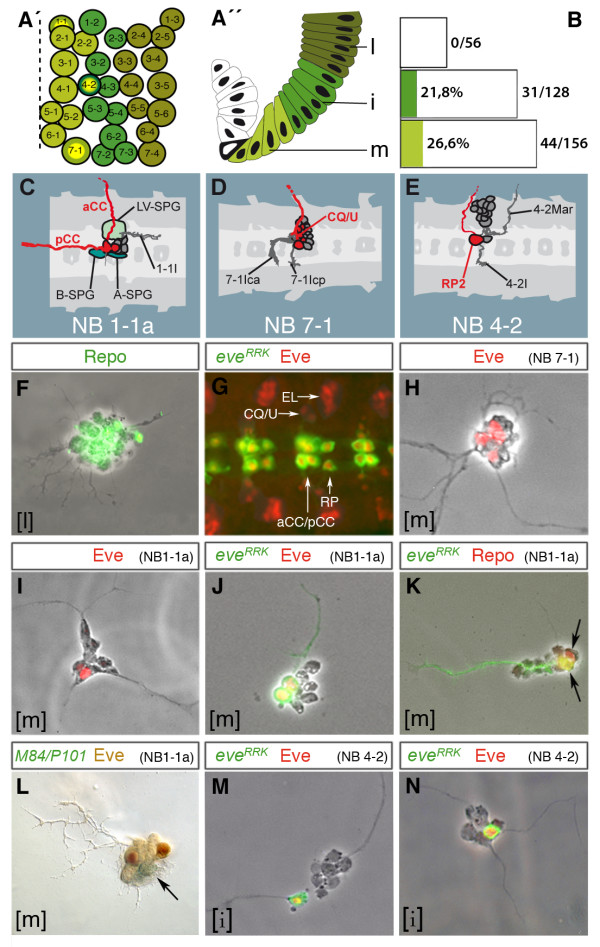
**Early specification and developmental autonomy of neuroectodermal progenitor cells**. **(A') **Neuroblast map of a truncal hemisegment at stage 10 (based on [1]). Anterior to the top; broken line marks midline. Medial (light green), intermediate (medium green) and lateral neuroblasts (dark green) delaminate from particular dorsoventral regions of the neuroectoderm as indicated by corresponding colour code in A''. **(A'') **Stage 7 neuroectoderm: m (medial, rows 1 to 4), i (intermediate, rows 5 to 9), l, (lateral, rows 10 to 15). Dorsal to the top, midline to the left (mesoderm, white; mesectoderm, black). **(B) **Proportion of cultured lineages comprising *eve*-GFP expressing cells. Progenitors derive from specific neuroectodermal domains (as indicated by colour code; compare A'') of the *eve-Gal4*^*RRK *^strain. **(C-E) **Semi-schematic presentation of embryonic lineages (horizontal views; anterior to the left; according to [3]), which include *eve*-expressing cells (marked in red; see text for further description). Progenitor cells NB1-1a (abdominal), NB7-1, and NB4-2 are highlighted with yellow spots in (A'). **(F) **Clone derived from a cultured lateral neuroectodermal progenitor (l), comprising glia in addition to neurons. **(G) **Ventral nerve cord (horizontal view; anterior to the left) of an *eve-Gal4*^*RRK*^/*UAS-mCD8::GFP *stage 15 embryo double-stained against CD8 (green; aCC/pCC, RP2) and Eve (red; lateral cluster (EL), CQ/U motoneurons, and co-expression in aCC, pCC, and RP2 (yellow nuclei)). **(H-N) **Clones derived from medial (m) or intermediate (i) neuroectodermal progenitors of wild-type (H,I), *eve-Gal4*^*RRK*^/*UAS-mCD8::GFP *(J,K,M,N) or M84/P101-*lacZ *embryos (L) as indicated. (**H,I**) Cones including four to five (H) and two (I) Eve-expressing cells, respectively. **(J) **Clone including two cells co-expressing CD8-GFP and Eve. (K) Clone including two Repo-positive glial cells (arrows) and two Eve-positive neurons (cell bodies out of focus)). **(L) **Clone including two Eve-expressing neurons (brown) and one to two *lacZ*-expressing cells with flat glia-like shape (green, arrow). **(M,N) **Clones including one prominent cell double stained against CD8 (green) and Eve (red). Cell sizes in (F,H-N) correspond to 5 to 6 μm. Markers and putative identities of cultured lineages are indicated on top of each picture, and their site of origin in the neuroectoderm at the bottom.

In the embryo each NB occupies a specific position in the NB layer corresponding to its site of delamination from the ectoderm (Figure 5A', A"), and it produces a specific lineage [1,3-5]. In order to test whether precursors already become firmly committed for specific NB fates in the neuroectoderm, and whether they are able to express their specific fate autonomously, we cultured single cells, which were taken (at stage 7) from well-defined dorsoventral positions in the abdominal neuroectoderm. Cells were removed from the neuroectoderm at a certain distance from the midline (1 to 4, 5 to 9, and 10 to 15 cell diameters/rows; the total dorsoventral dimension of the neuroectoderm was approximately 15 cell diameters; Figure 5A"). They were individually grown in culture for up to 20 hours, and their lineages stained against cell specific markers.

First, we analyzed the ability of cultured neuroectodermal progenitor cells to produce glial progeny by staining with the glial specific anti-Repo antibody. Out of 106 clones deriving from ventral progenitor cells of neuroectodermal rows 1 to 4, 27 (26%) included glial cells in addition to neurons. The number of glia in these clones varied between one and four cells (as some of the glia stay in close association with other components of their lineages, and due to their flat shape assessment of their exact number is often difficult). In the embryo the only glial progenitor delaminating from this region in abdominal segments is the neuroglioblast NB1-1, which produces three glial cells in addition to neurons (see below). The rather high frequency of this type of clone obtained in culture (26%) is probably due to the fact that cells were removed from the neuroectoderm at an early stage, when proneural clusters for early delaminating NBs (S1, S2) [1,3] are in place while those for late delaminating NBs (S3 to S5) may still have to be established (see also distribution of clonal frequencies below). In the embryo only four out of about nine ventral NBs delaminate during S1/S2, and NB1-1 is one of them (corresponding to 25%). Cultured progenitor cells deriving from the most dorsal sector of the neuroectoderm (rows 10 to 15; n = 332) gave rise to clones that stained positive for Repo in a higher number of cases (37%; 122 clones). Of these Repo-positive clones, 104 (85%) were mixed clones comprising 1 to 6 glial cells in addition to neurons (Figure 5F), whereas 18 clones (15%) consisted of glial cells exclusively (data not shown). These purely glial clones generally consisted of either only two cells (nine clones) or eight cells (six clones). Thus, the percentage of cultured clones comprising glial cells depends on whether precursors were taken from ventral or dorsal sites of the neuroectoderm. This resembles the situation *in vivo*: in the embryo, almost all glial progenitor cells (except abdominal NB1-1 and thoracic NB2-2) derive from the most dorsal sector of the neuroectoderm (rows 10 to 15), among them four neuroglioblasts producing mixed lineages (NB1-3, NB2-5, NB5-6, NB7-4) and two glioblasts (NB6-4a, GP). The abdominal NB6-4a glioblast gives rise to only two glial cells, and the GP produces seven to nine glial progeny [3,4].

Second, we analyzed lineages that give rise to cells expressing the marker *even-skipped *(*eve*). NB1-1 and NB7-1 belong to the subset of medial NBs (Figure 5A, C, D) and delaminate from the most ventral part of the neuroectoderm (one to three cell diameters apart from the ventral midline according to DiI labelling [3]). NB4-2 belongs to the subset of intermediate NBs and delaminates from a ventrolateral site of the neuroectoderm (five to nine cell diameters apart from the ventral midline; Figure 5A, E). In the embryo, abdominal NB1-1 produces a clone consisting of three subperineurial glial cells, one motoneuron (aCC), one interneuron projecting anteriorly (pCC), and a small cluster of four to six interneurons forming a fascicle projecting posteriorly (Figure 5C) [36]. aCC and pCC express *eve *[37]. The NB7-1 lineage (Figure 5D) consists of 16 to 22 neurons, including 5 *eve*-expressing cells (CQ/U neurons [3,37,38]). NB4-2 gives rise to 8 to 14 interneurons and two motoneurons, one of which (RP2) expresses *eve *[3,38] (Figure 5E). The pattern of Eve-expressing cells in the ventral nerve cord of a stage 15 embryo is shown in Figure 5G.

When culturing cells from the ventral-most neuroectoderm (rows 1 to 4) and staining with an anti-Eve antibody (n = 532), we obtained 199 clones (37.4%) with *eve*-expressing progeny. Of these clones, 33% included 3 to 7 (Figure 5H) and 67% included 1 to 2 Eve-positive cells (Figure 5I). With regard to their site of origin and numbers of *eve*-expressing progeny, these clones appear to reflect characteristics of the NB1-1 and NB7-1, respectively.

To test this in more detail, we used the line *eve-Gal4*^*RRK *^[39], which expresses Gal4 exclusively in the NB1-1-derived aCC and pCC and in the NB4-2-derived RP2 motoneuron [40]. We used this line to drive expression of green fluorescent protein (GFP; *UAS-mCD8::GFP *[41]) in these cells (Figure 5G). Embryos carrying these constructs were used as donors for neuroectodermal cells. Out of 165 clones obtained from individually cultured neuroectodermal cells of rows 1 to 4, 44 (26.6%; Figure 5B) included 2 cells (79.5% of the 44 clones; Figure 5J), 1 cell (11.5%), and 3 cells (9%) expressing *eve*^*RRK*^; as reflected by a prominent axonal fascicle, these cells are neurons. The total number of cells within these clones generally varied between 8 and 14. In some clones *eve*-expression in these cells was confirmed by double staining with the anti-Eve antibody (Figure 5J). Furthermore, we double-stained 11 of the mCD8::GFP-positive clones from rows 1 to 4 against the glial marker Repo and found two Repo-positive cells in six (Figure 5K), and one Repo-positive cell in one of these clones. We also cultured single neuroectodermal cells of rows 1 to 4 from the enhancer trap strain M84/P101 [42]. This line expresses *lacZ *specifically in a set of subperineurial glial cells, including those produced by abdominal NB1-1 [36]. Figure 5L shows a clone double-stained against Eve and β-galactosidase that includes two cells expressing Eve and one to two cells expressing β-galactosidase and exhibiting glial morphology. Since abdominal NB1-1 is the only precursor generating *eve*-expressing cells in addition to glia, the expression of both markers *in vitro *is indicative for the NB1-1 fate (Figure 5C).

Finally, 31 out of 142 clones (21.8%; Figure 5B) produced by progenitors from neuroectodermal rows 5 to 9 included 1 (93.5% of the 31 clones; Figure 5M, N) or 2 (6,5%) *eve*^*RRK*^-positive cells in addition to 6 to 12 *eve*-negative cells. This reflects characteristics of the NB4-2 lineage *in situ *(Figure 5E). Furthermore, the *eve*-positive cell develops a prominent fibre projecting separate from other clonal fibres. Finally, this cell tends to move a short distance apart from the clonal cell cluster (Figure 5M), as is the case for RP2 in the embryonic NB4-2 lineage (Figure 5E). In contrast, none of the progenitors taken from the most dorsal site of the neuroectoderm (rows 10 to 15; n = 56; Figure 5B) gave rise to progeny expressing the marker *eve*^*RRK*^, as is the case *in situ*.

Taken together, these data indicate that presumptive NBs have already acquired a high degree of commitment in the neuroectoderm, and are able to cell-autonomously express specific characteristics of their lineages when grown in primary culture.

## Discussion

### Culturing individual neural precursors of defined spatial and temporal origin

*Drosophila *primary cultures have been used for decades to investigate various aspects of neural development and function. The morphological, physiological and molecular characterization of primary neural cultures revealed that their developmental and physiological properties mirror a great number of characteristics in the intact organism [31,43-57]. Since large numbers of cells from dissociated early embryos or a NB-enriched fraction of cells were cultured in most of these experiments, the sites of origin of the cells in the embryo and their precise developmental stage were not known. Under these conditions, comparisons of developmental capacities of specific types of precursors *in situ *and *in vitro *are very limited, if at all possible.

We have therefore established a means to remove progenitor cells from specific sites (as defined by morphological landmarks and the availability of a detailed fate map [58]) of precisely staged embryos and grow them individually in culture. So far, we have used this method to study the intrinsic component in the determination of neural versus epidermal cell fate as a function of the distribution of progenitor cells along the dorsoventral axis of the ectoderm [31], and to study the cell-autonomous component in the expression of ionic currents by neurons derived from CNS midline precursors [54]. In these experiments, however, we did not distinguish and compare between specific types of CNS lineages.

Here we studied in single cell cultures the development and composition of lineages generated by CNS midline precursors and by presumptive NBs taken at the early gastrula stage from specific dorsoventral domains of the truncal neuroectoderm. By comparison with the previously described CNS lineages *in situ *[3-5,22], this approach allows cell-autonomous properties versus the requirements for extrinsic signals during lineage development of specific subpopulations of CNS progenitor cells to be uncovered for the first time.

### Non-cell-autonomous control of proliferation and cell fate determination within CNS midline lineages

The mesectodermal midline cells are initially specified during the blastoderm stage by the master regulator gene *single-minded *(*sim*), which is required for subsequent development of all midline cells [59]. Transplantation experiments revealed that premitotic mesectodermal cells (early gastrula, stage 7) are firmly committed to form midline progenitors and to occupy/maintain a midline position in the developing CNS [11]. Individually cultured mesectodermal precursor cells exhibit cell-autonomous developmental capacities with regard to the expression of morphological characteristics of neuronal and glial cell fates, and to programmed cell death. Furthermore, as previously shown for midline neurons, the expression of voltage-gated potassium currents appears to be cell-autonomous [54]. These autonomous properties represent rather general characteristics that apply for the entire ventral midline primordium.

In contrast, the establishment of specific characteristics and diversity among midline lineages does not appear to be a cell-autonomous property of midline progenitors. The clones we obtained from individually cultured midline progenitors significantly differed from midline lineages *in situ *with regard to proliferation (clone size distribution, no asymmetric divisions) and the expression of molecular markers (co-expression of neuronal and glial markers). Thus, midline progenitors grown in isolation lose their ability to properly control their proliferation and clear definition and/or separation between neuronal and glial fate. This suggests that determination of specific aspects of their fate requires extrinsic inductive signals. A possible requirement for signalling among components of different midline lineages has been discussed previously as a means for matching variable numbers of midline progenitors (six to eight cells per segment) with the final population of postmitotic progeny cells [22]. Bossing and Brand [23] have shown that cell fates within the CNS midline are determined after the precursors divide by Wingless and Hedgehog signalling acting on their daughter cells. Furthermore, Wheeler *et al*. [15] reported recently that, after the stage 8 division, Notch signalling promotes midline glia and MNB cell fate and is also required for asymmetric daughter cell fate in particular midline lineages. However, it is not clear as to how far these signals act among lineage-related and/or non-related progeny cells, and little is known about influences on midline precursor cell fate before they enter mitosis at stage 8. In our experiments, individual pre-mitotic midline precursors have been removed from the embryo at stage 7. Therefore, exchange of signals within their lineages should still be possible in culture, whereas those coming from other lineages would be lacking (the same is true for the NB lineages in culture; see below). The characteristics of the cultured clones reveal that cell autonomous properties of midline precursor cells and signalling among clonally related progeny cells are not sufficient for normal control of their development. This is indicative for the existence of inductive signals in the embryo that are acting early within the mesectoderm and/or coming from neighbouring primordia (neuroectoderm, mesoderm). The nature of these signals remains to be clarified.

### Development of presumptive neuroblasts shows a high degree of cell autonomy

In the embryo each of the approximately 30 NBs per truncal hemisegment expresses a characteristic set of molecular markers [1,37] and produces a unique cell lineage [3-5]. Superimposition of segment polarity and dorsoventral patterning gene activities in the neuroectoderm can explain how each descending NB acquires an individual fate (reviewed in [6,7]). Heterotopic and heterogenetic transplantation experiments have previously revealed a high degree of commitment of early neuroectodermal cells for ventral NB fates [11] and for segmental specificity [10,60]. Furthermore, heterochronic transplantations have shown that the specification of temporal subsets of NBs occurs under the control of stage-specific inductive signals acting in the neuroectoderm [9].

Our *in vitro *experiments indicate a high degree of cell-autonomy of early neuroectodermal cells in generating specific types of lineages. As individually cultured progenitor cells were devoid of signals coming from other primordia (neuroectoderm, mesoderm) or other lineages, their early exposure to positional cues within the neuroectoderm appears to be sufficient for specification and subsequent development of characteristic features of particular NB lineages, like NB1-1. Accordingly, NBs cultured after their delamination in cell suspensions of approximately 5-hour-old embryos develop lineages showing the same temporal transcription factor expression windows (Hunchback → Pou-homeodomain proteins 1 and 2 → Castor → Grainyhead) as in the embryo [44]. On the other hand, delaminated NBs require extrinsic signals from the overlying neuroectoderm during interphase to regulate spindle position and apical protein localization [12]. However, since interphase protein localization appears to be unnecessary for subsequent protein localization and unequal NB cytokinesis at mitosis [43], individual NBs *in vitro *divide asymmetrically in a normal stem cell mode, producing a chain of progeny cells that inherit differential cell fates.

## Conclusion

We have analyzed the development and composition of lineages generated by individually cultured mesectodermal CNS midline precursors, and by presumptive NBs taken from specific dorsoventral domains of the truncal neuroectoderm. Comparison of the clones generated by neural precursors of defined spatial and temporal origin *in vitro *with the well-described characteristics of the lineages *in situ *uncovers cell-autonomous properties versus the requirements for extrinsic signals during development of the respective progenitor cells. Our experiments demonstrate that the isolated cells express characteristics of the progenitor type appropriate to their site of origin in the *Drosophila *embryo. However, the two sets of CNS progenitor cells exhibit different degrees of cell-fate commitment at the pre-mitotic and pre-delamination stage. Presumptive NBs, once specified by positional information in the neuroectoderm, show a higher degree of autonomy regarding the generation of their lineages as compared to mesectodermal midline progenitors.

## Materials and methods

### *Drosophila *strains

We used Oregon R wild-type flies and the following enhancer trap lacZ, Gal4 and UAS lines: M84, P101, AA142 [42]; *eve-Gal4*^*RRK *^[39,40]; *repo-Gal4 *[61]; *eagle*-*Gal4 *(*MZ360*) [62]; *UAS-mCD8::green fluorescent protein *(*UAS-mCD8::GFP *[41]; Bloomington Stock B-#5130), and *UAS-lacZ *[63].

### Single cell cultures

#### Culture medium

We used Schneiders medium for culture medium [64-66]. Non heat-inactivated foetal calf serum was added to 10%. After addition of serum, the medium was kept for 3 days at 25°C, then insulin (200 ng/ml) was added, and pH adjusted to 6.8 to 6.9.

#### Isolation and culturing of individual neural progenitor cells

Embryos at the blastoderm stage were washed in 70% ethanol, dechorionated, mounted in an appropriate orientation on a cover-slip coated with glue, desiccated and covered with fluocarbon oil. Embryos were selected as donors at about stage 7 to 8 (stages according to [67]), when the midline precursors are clearly distinguishable. A capillary (provided with an approximately 45° bevel and an inner tip diameter of 12 to 13 μm for the removal of midline progenitors, and a 10 to 12 μm diameter for the removal of neuroectodermal progenitor cells) was introduced into the embryo along the longitudinal axis, and three to six cells from the ventral midline or from specific dorsoventral cell rows of the neuroectoderm (as counted from the ventral midline) were removed. Cells were cultured as previously described [31]. Briefly, a single cell was released from the capillary and placed centrally onto a clean, sterile glass coverslip in a drop (20 to 30 μl) of sterile culture medium. In cases when more than one cell was released into the medium, they were deposited individually with large separation distances in order to prevent cell interactions. The cover slip (greased at its fringes) was then sealed onto a small, sterilized culture vessel [68]. The vessel was turned upside down, placed over the coverslip and pressed down to seal the chamber tightly. The drop of medium adhered to both sides to form a column in the centre of the chamber. Cultures were kept for 16 to 20 h in the dark in an incubator at 26°C, or their development was continuously traced at room temperature (approximately 22°C) by time-lapse recordings as previously described [31].

### Staining procedures and antibodies

Cells were fixed and stained as previously described [31]. Cells were stained for β-galactosidase expression with rabbit-anti-β gal antibodies (1:5,000; Cappel-Promega, Mannheim, Germany) or using X-Gal staining. Apoptotic cells were detected by TUNEL staining (*in situ *Cell Death Detection kit, Roche Diagnostics, Mannheim, Germany) or by antibody staining against rabbit anti-human activated Caspase 3 (1:50; Cell Signalling Technology, Danvers, MA, USA). Additionally, the following primary antibodies were used: mouse anti-Even-skipped (1:2; Developmental Studies Hybridoma Bank, University of Iowa, IA, USA), rabbit anti-GFP (1: 250; Torrey Pines Biolabs, East Orange, NY, USA), mouse anti-HRP (1:20; Dako, Hamburg, Germany), rabbit anti-Odd-skipped (1:5,000; James Skeath, Washington University School of Medicine, St Louis, MO, USA), rabbit anti-Repo (1:200) [32], rat anti-serotonin (1:50; Accurate, Westbury, NY, USA), and rat anti-TTK (1:1,000) [26]. The secondary antibodies used were: anti-rat-fluorescein isothiocyanate (FITC), anti rabbit-FITC, anti mouse-Cy3, anti mouse-alk. Phosphatase, anti mouse-HRP, and anti rabbit-HRP (1:300; all Jackson ImmunoResearch, Suffolk, UK). For DAB stainings, the ABC kit from Vectastain (Burlingame, CA, USA was used.

Colour images were made using a Zeiss Axioskop 2 microscope.

## Competing interests

The authors declare that they have no competing interests.

## Authors' contributions

KL carried out the cell culture studies and created the figures. GMT conceived of the study and wrote the manuscript.

## Supplementary Material

Additional file 1 **Differentiation and cell death in a cultured midline lineage**. Time-lapse movie showing a midline lineage developing *in vitro*. Three post-mitotic progeny cells exhibiting glial morphology are generated. One of these cells undergoes cell death (disintegration into several particles), whereas the other two convert their shape to assume neuronal morphologies (cell bodies round up and form long fibre projections). Real time is indicated at the bottom. Selected frames are shown in Figure 2B. Click here for file

Additional file 2 **Differentiation and cell death in a cultured midline lineage**. Time-lapse movie showing a midline lineage developing *in vitro*. Two divisions lead to three progeny cells exhibiting glial morphology. One of these cells moves apart from the others and undergoes cell death (disintegration into several particles). Real time is indicated at the bottom. Selected frames are shown in Figure 2A. Click here for file

Additional file 3 **Comparison of clone sizes obtained *in vitro *and *in situ *from progenitors of the ventral and dorsal half of the neuroectoderm**. Comparison of clone sizes obtained *in vitro *and *in situ *from progenitors of the ventral and dorsal half of the neuroectoderm. Click here for file

Additional file 4 **Development of an individually cultured neuroectodermal progenitor cell**. Time-lapse movie showing the development of a cultured progenitor cell that originated from the most ventral domain of the neuroectoderm. The generation of the first two ganglion mother cells (GMC1 and GMC2) by asymmetric divisions of the neuroblast and the symmetric division of GMC1 (taking place after GMC2 is born) into two postmitotic progeny cells (P) are indicated in the film. Assignment of further divisions is difficult due to the dense, three-dimensional arrangement of progeny cells. Note that during maturation of the clone (after approximately 23:15) a prominent pair of equally sized cells (which seem to be the two progeny of GMC1) moves a short distance apart from the cell cluster (along a fibre bundle) to later join the cluster again. Real time is indicated at the bottom. Selected frames and a schematic of the early divisions are shown in Figure 3. Click here for file
