# Supplementary material for: Single cell cultures of Drosophila neuroectodermal and mesectodermal central nervous system progenitors reveal different degrees of developmental autonomy
Source: Neural Dev. 2009 Aug 3;4:30. doi: 10.1186/1749-8104-4-30 (PMC2736940; doi:10.1186/1749-8104-4-30)
Supplement: Additional file 3 — Comparison of clone sizes obtained in vitro and in situ from progenitors of the ventral and dorsal half of the neuroectoderm. Comparison of clone sizes obtained in vitro and in situ from progenitors of the ventral and dorsal half of the neuroectoderm. [file 1749-8104-4-30-S3.doc]

**Table I. Comparison of clone sizes obtained *in vitro* and *in situ* from progenitors of the ventral and dorsal half of the neuroectoderm**

| **Clone**  **sizes** | | **1** | | **2** | **3** | **4** | **5** | **6** | | **7** | **8** | **9** | **10** | | **11** | **12** | | **13** | **14** | | **15** | **16** | | **17** | **18** | **>18** |
| --- | --- | --- | --- | --- | --- | --- | --- | --- | --- | --- | --- | --- | --- | --- | --- | --- | --- | --- | --- | --- | --- | --- | --- | --- | --- | --- |
| **Vental half of neuroectoderm** | ***in vitro^#^*** | **-** | | **+** | **+** | **+** | **+** | **+** | | **+** | **+** | **+** | **+** | | **+** | **+** | | **+** | **+** | | **+** | **+** | | **-** | **+** | **+** |
|  | ***in situ^§^*** |  | | MP2 |  | | | | | | | | | | | | | | | | | | | | | |
|  |  |  | | NB 5-1 | | |  | | | | | | | | | | | | | | | | | | | |
|  |  |  | | | | | NB 2-1 | | | | | | | |  | | | | | | | | | | | |
|  |  |  | | | | | | | | | NB 7-2 | | | | | | | | |  | | | | | | |
|  |  |  | | | | | | | | | NB 6-2 | | | | | | | | | | | |  | | | |
|  |  |  | | | | | | | | | | NB 1-1a | | | |  | | | | | | | | | | |
|  |  |  | | | | | | | | | | NB 5-3 | | | | | | | | | |  | | | | |
|  |  |  | | | | | | | | | | | NB 3-1a | | | | |  | | | | | | | | |
|  |  |  | | | | | | | | | | | NB 6-1 | | | | | | | | | | |  | | |
|  |  |  | | | | | | | | | | | NB 4-2 | | | | | | | | | | |  | | |
|  |  |  | | | | | | | | | | | NB 3-2 | | | | | | | | | | | | |  |
|  |  |  | | | | | | | | | | | | | | NB 4-1 | | | | | | | | | |  |
|  |  |  | | | | | | | | | | | | | | | | | | | | NB 7 -1 | | | | |
|  |  |  | | | | | | | | | | | | | | | | | | | | | | | | NB 1-2 |
| **Dorsal half of neuroectoderm** | ***in vitro^#^*** | **-** | **+** | | **+** | **+** | **+** | | **+** | **+** | **+** | **+** | **+** | | **+** | **+** | | **+** | **+** | | **+** | **-** | | **+** | **+** | **+** |
|  | ***in situ^§^*** |  | 6-4a | |  | | | | | | | | | | | | | | | | | | | | | |
|  |  |  | | | NB 7-3 | | | |  | | | | | | | | | | | | | | | | | |
|  |  |  | | | | NB 5-4a | | |  | | | | | | | | | | | | | | | | | |
|  |  |  | | | | | | | NB 5-5 | | | | |  | | | | | | | | | | | | |
|  |  |  | | | | | | | | NB 2-4a | |  | | | | | | | | | | | | | | |
|  |  |  | | | | | | | | NB 5-6a | | | | |  | | | | | | | | | | | |
|  |  |  | | | | | | | | | NB 4-4 | | | | | |  | | | | | | | | | |
|  |  |  | | | | | | | | | NB 4-3 | | | | | | | |  | | | | | | | |
|  |  |  | | | | | | | | | | NB 1-3 | | | | | | |  | | | | | | | |
|  |  |  | | | | | | | | | | | NB 3-3 | | | | | |  | | | | | | | |
|  |  |  | | | | | | | | | | | | | | NB 7-4 | | | | | | | | | | |
|  |  |  | | | | | | | | | | | | | | | | | | | | NB 2-5 | | | | |
|  |  |  | | | | | | | | | | | | | | | | | | | | | | | | NB 3-5 |

^#^ clones sizes generated by cultured cells are marked (+, in all cases n > 5); exact counting was difficult for clones consisting of more than18 cells.

^§^ *in situ* data according to [3,4]. Range of clones sizes (grey bars) is indicated for each NB, and reflects variability and (for larger clones) imprecise counting
